# Operative treatment and outcome of unstable distal radial fractures using a palmar T-miniplate at a non-specialized institution

**DOI:** 10.1007/s11751-013-0170-y

**Published:** 2013-08-06

**Authors:** E. Skouras, Y. Hosseini, V. Berger, K. Wegmann, T. C. Koslowsky

**Affiliations:** 1 Klinik und Poliklinik für Orthopädie und Unfallchirurgie, Universitsklinikum Köln, Kerpener Straße 62, 50937 Cologne, Germany; 2 Department of Surgery, St. Elisabeth-Hospital, Werthmannstraße 1, 50935 Cologne, Germany

**Keywords:** Distal radial fractures, T-miniplate osteosynthesis

## Abstract

Treatment options for displaced distal radial fractures are still a controversial topic of discussion. Although good results for the palmar plating of high-volume centers have been published, evidence of its successful use in smaller institutions is still lacking. We report the clinical and radiological results of the treatment for 84 distal radial fractures with a single 2.4-mm T-miniplate in an institution performing <30 procedures per year. According to the AO classification system, there were 30 A, 5 B, and 49 C fractures with a patients mean age of 64 years. After a minimum of 12-month follow-up, we found very good and good results according to the Gardland and Sarmiento scores and a DASH of 5.6. Only five patients were classified as having a moderate outcome. A remaining intra-articular step-off of more than 1 mm was seen in 15 patients. In a comparison of grip strength between the injured and uninjured hands, we saw a difference of 6.8 % less on the injured side. We saw two instances of tendon rupture and one of tendon irritation due to prominent dorsal screws and necessitating revision surgery. Flexor tendon irritation was noted in one patient, requiring a second operation. Modern treatment for distal radial fractures can be performed successfully and with good clinical outcome in smaller institutions. Based on the high and increasing incidence of distal radial fractures, there is no need to transfer these patients into high-volume centers.

*Level of evidence* Case study, Level IV.

## Introduction

Distal radial fractures seem to be the most common fracture entity currently seen in accident and emergency units, with an annual estimated incidence of 36.6 women/10,000 and 8.9 men/10,000 per year [1]. A significantly growing elderly population with a markedly increasing life expectancy may increase the fracture incidence by a further 50 % by the year 2030 [2]. Non-operative treatment using a plaster cast is usually chosen for non-displaced fractures. A stable reduction in displaced fractures may also be treated non-operatively [3]. Unstable and displaced radial fractures are treated operatively. Besides stability and displacement, intra-articular or extra-articular fracture type may also be important for the decision. The ideal method for surgical management of these fractures has been a controversial topic of discussion, and numerous procedures are available. Percutaneous Kirschner wire fixation, joint-bridging and non-joint-bridging external fixation, or a combination of both can be used successfully [4–6]. The palmar locking plate has recently become popular [5, 7, 8] for treatment for distal radial fractures, and good to excellent clinical results have been published [8–11]. In fact, there are a number of studies demonstrating good results in the treatment for distal radial fractures with palmar locked angle plates at a large, specialized institution. The limited number of specialized institutions coupled with the increasing number of distal radial fractures may be leading to a lack of treatment capacity of this injury. The purpose of this prospective study was to evaluate the subjective and objective outcome after operative treatment for distal radial fractures using a palmar locked angle miniplate (Koenigsee, 2.4 mm T-miniplate) by a smaller, non-specialized institution to evaluate the necessity for referral to high-volume services.

## Patients and methods

This study was performed at a hospital with a non-specialized trauma service with the healthcare level of basic trauma and reconstructive surgery. Over a period of 3 years (2005–2007), a total of 82 patients with 84 distal radial fractures (mean age 64 years (18–94 years); 15 males and 67 females) were followed prospectively. Preoperative work-up included a clinical examination and standard two-plane X-rays. After initial sandwich casting, the operation was performed as an elective procedure with a mean of 4 days (injury day to day seven) after trauma, except for those patients with open fractures (four patients) and/or neurological affection (one patient). Besides these emergency criteria, indications for surgical intervention were the following: radial shortening of more than 3 mm; dorsal comminution; dislocation of more than 20° in extra-articular fractures; or an intra-articular step-off of more than 2 mm. According to the AO classification system, there were 30 A (eight A2, 22 A3), 5 B (one B1, one B2, and three B3) and 49 C (18 C1, 24 C2, and seven C3) fractures. A total of 77 patients were right-handed and five were left-handed. Thirty-six patients had injured the dominant hand; 46 patients had injured the non-dominant hand. Two patients had injured both hands.

There were two distal ulna fractures, which were treated additionally with a descending intramedullar elastic nail. Two patients had suffered proximal femur fractures that were treated simultaneously by prosthetic replacement or a Y-nail, respectively.

**Fig. 1:**
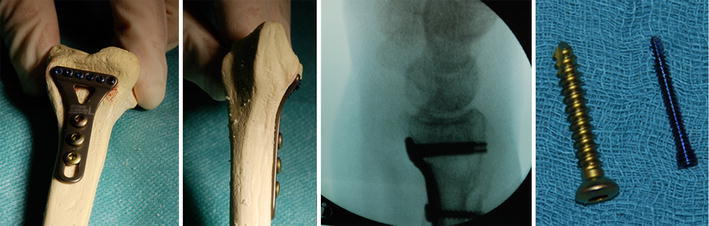
A fluoroscopy of the dimensions of the palmar miniplate on a sawbone model. The right side of the picture shows the self-cutting 3.5-mm screws for the long bar of the plate. Six smaller 2.2-mm locked angle miniscrews have been placed at the distal part of the plate, each addressing as many fracture fragments as possible

All patients were treated by two surgeons with a 3 year of special training in trauma and orthopedic surgery with the same type of implant: the 2.4-mm T-miniplate (Koenigsee Implantate, Aschau, Germany) with either three or four holes in the shaft bar for conventional 3.5-mm cortical screws and six 2.2-mm fine-threaded locked angle miniscrews for the diagonal distal bar. Figure 1 shows the dimensions of the plate and screw placed on a sawbone model via an intraoperative X-ray. Operative procedures were performed using a tourniquet, and a standard distal Henry approach was used for fracture exposure. In extra-articular fractures, distal screws were placed parallel to the joint surface, and reduction was performed using the “lift technique” [12]. Intra-articular fractures were reduced beginning with the distal radioulnar fragment, where a guiding K-wire was placed precisely into the radio-ulna corner with the use of an image intensifier, again parallel to the radiocarpal and radioulnar joint surface. Then, a miniplate with the locked angle guiding drill holder was placed over the K-wire. A stepwise reduction and fixation of the joint block was performed with the locked angle 2.2-mm miniscrews until the initial K-wire was exchanged for another miniscrew. As the next step, the whole joint block was reduced and reaffixed to the shaft using the “lift technique.” Here, special care was taken with the reduction in the distal radioulnar joint. Finally, the pronator quadratus muscle was reaffixed as far radial as possible, at least enough to protect the plate from the flexor pollicis longus tendon. Postoperative casting was performed for 6 days to initiate regular wound healing. Hospitalization was 6 days in mean (1–25 days). Then, functional treatment without casting or splinting with additional physiotherapy was performed twice weekly. X-rays were taken on days two, seven, and 42 after the operation and at the time of follow-up. Degenerative changes were detected according to Knirk and Jupiter [13].

Follow-up was performed by the first and second authors, MS and YH, including a radiological and clinical work-up. The radiological work-up was performed on a PACS system and included the congruency of the joint surface, dorsal angulation, and loss of dorsal angulation. Radial shortening was measured as a vertical distance between the ulna border of the distal radius and the most distal point of the ulna head [14]. Malunion was defined as a dorsal angle less than zero degrees, a palmar angle <15°, a carpal malalignment [15], a distal radial shortening of more than 3 mm, or a combination of these parameters [8].

The clinical follow-up included a standardized examination of the injured and contralateral side. Range of motion of the wrist was determined on the frontal and sagittal planes, and pronation and supination were measured according to the neutral 0 method with a standard goniometer. To determine the functional results, we used the Sarmiento [16], Gardland [17], and DASH [18] scores.

Grip strength in Newtons was measured on both sides using a computer-assisted hydraulic hand goniometer (Vernier Software & Technology^®^, Beaverton, Oregon).

Results were analyzed with a Student’s *t* test, and significance was granted for *p* < 0.05. For patient with double-side fractures, only one clinical score was performed.

## Results

**Fig. 2:**
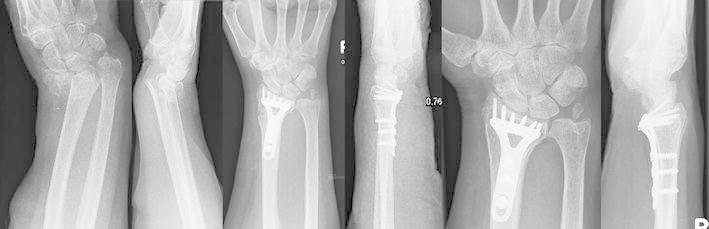
A complex AO C2 distal radial fracture and its reconstruction with a locked angle miniplate. The clinical result shows a free and unlimited function of the wrist. The patient only complains a prominent ulnar head without pain due to the nonunion of the ulnar styloid and minimal ulnar plus. The increased radial inclination was asymptomatic

The clinical and radiological data are summarized in Tables 1, 2, 3, and 4. At the time of follow-up, clinical results were very good or good according to the Gardland and Sarmiento scores with a DASH score of 5.6. Only five patients were classified as having a moderate outcome. Function was almost unlimited. We saw an anatomical reduction in the distal radius including the joint surfaces and, in Typ A Fractures, in the metaphyseal area in 58 fractures. A remaining intra-articular step-off of more than one mm was seen in 15 fractures at the first postoperative X-ray, whereas four patients had an intra-articular step-off in the radiocarpal joint and 11 in the distal radioulnar joint; arthritic changes were seen in 42 fractures at the time of follow-up. Compared to the first postoperative X-ray, we saw a loss of radial length of 2 mm [from −2.41 mm (−7–5 mm) to −0.23 (−4–6 mm)] at the time of follow-up. There was no significant loss of palmar tilt and radial inclination (see Table 1). In comparing grip strength between the injured (78.5 N; 8.9–216 N) and uninjured hands (84.2 N; 24.9–236.2 N), we saw a difference of 6.8 % less strength on the injured side. At the time of follow-up, we could identify a total number of two tendon ruptures and one case of tendon irritation due to prominent dorsal screws, leading to revision surgery. Flexor tendon irritation was noted in one patient, necessitating a second operation. The two tendon ruptures were treated with an indicis proprius transfer. No complex regional pain syndrome (CRPS) and no infection were seen in this study. Figure 2 shows a typical prä and postoperative X-ray of a AO C2 distal radial fracture treated with a miniplate.

**Table 1:** Radiological results at the time of follow-up

	At first X-ray post OP	At follow-up
Palmar tilt	11.75° (5°–15°)	11.60° (5°–14°)
Radial inclination	20.09° (15°–29°)	20.98° (15°–29°)
Radial shortening	−2.41 mm (−7–5 mm)	−0.23 mm (−4–6 mm)
Joint surface incongruency (radiocarpal > 1 mm)	4 × >1 mm	
Joint surface incongruency (radioulnar > 1 mm)	11 > 1 mm

**Table 2:** Arthritic changes detected at the time of follow-up in the postoperative X-ray

Arthritic changes	Grade 0	Grade 1	Grade 2	Grade 3
Initial post OP X-ray	49	26	9	0
At follow-up	42	33	9	0

**Table 3:** The clinical scores obtained at the time of follow-up

Clinical data	Excellent	Good	Moderate	Bad
Sarmiento score	67	15	0	0
Gardland score	52	25	5	0

**Table 4:** The functional results obtained at the time of follow-up

	Injured side mean (min–max)	Uninjured side mean (min–max)
Supination (°)	86.19° (20°–90°)	88.1° (40°–90°)
Pronation (°)	87.67° (45°–90°)	88.5° (40°–90°)
Extension (°)	56.72°(30°–70°)	60.3° (40°–80°)
Flexion (°)	62.5° (30°–90°)	68.1° (55°–90°)
Radial abduction (°)	25.29° (10°–35°)	29.6° (20°–40°)
Ulna abduction (°)	34.16° (15°–50°)	38.9° (20°–55°)

## Discussion

A significantly growing population of elderly patients and their increasing life expectancy will lead to an increase in osteoporotic fractures. Besides fractures of the proximal femur, proximal humerus, and vertebral column, distal radial fractures play an important role in the medical treatment of this age group. The patients treated by our hospital with an incidence of 49 (out of 84) AO type C fractures and a mean age of 64 years are comparable to those reported by other institutions [8, 10]. The high incidence of osteoporosis in this age group and the sex distribution of 67 women to 15 men may be the reason for the high incidence of complex fracture patterns: Figl et al. [10] report a number of 34 (out of 85) AO type C fractures, and their incidence has increased from 31 (out of 46) C type fractures as reported by Wei et al. [5] to 71 % as reported by Jupiter et al. in 2010 [9]. The treatment for distal radial fractures is a controversial topic of discussion. Despite evidence that an unsatisfactory radiological outcome does not necessarily predict deficient clinical results after non-operative treatment for distal radial fractures in senior citizen patients [19], the current literature on this fracture pattern shows a trend toward operative treatment [20]. Operative treatment options range from isolated pin fixation to external fixation to locked angle plate fixation [6, 9, 21]. Isolated pinning was reported by Goften and Liew [6]. The authors report this method to be effective for fractures that are too unstable for non-operative treatment. In the recent literature, several authors compare external fixation to palmar locking plate fixation.

Wei et al. [5] published a DASH of 18 12 months after external fixation compared to five in the volar locking plate group. A standard deviation of 14 and four, respectively, led to an insignificant difference between these two groups. Grip strength after 12 months was 18 kg (external fixation) versus 16.9 kg (volar plating), again without any significant differences, including the same ranges of motion in the two fixation groups. In contrast to these almost identical results at the end point of the study after 1 year, the authors saw favorable results in the volar plating groups just 3 months after fixation: in other words, the volar plating group reached their final good results earlier.

These results were supported by Rizzo et al. [22]: they found similar grip strength and range of motion after a follow-up of 29 months in a total of 41 patients, whereas DASH and radiographic outcomes were better in the volar plating group compared to the external fixation and pinning group. They concluded that volar plating seemed to be favorable to external fixation.

On the other hand, Abramo et al. [4] did not find any subjective difference between volar plating and isolated external fixation, but grip strength and ROM were better in the volar plating group 1 year after surgery.

Although the results of objective and subjective outcomes might be confusing regarding these two methods of treatment, at least there is evidence that in the best cases, external fixation is comparable to if not worse than volar plating [4, 22]. The good results achieved in these studies with volar plating compared to other implants are also supported by isolated clinical and radiological outcome studies: Knight et al. [8] report good clinical results after following 40 patients for 59 weeks with a mean DASH of 23, and Figl et al. [10] report a grip strength of 65 % on the contralateral ineffective side with a DASH of 25. They saw a volar loss of reduction of 2° from the initial postoperative X-ray to the study end point and a loss of radial length of one mm a mean of 59 weeks after surgery. Interestingly, they saw carpal malalignment in six of their 40 patients. The authors explained this fact with a high grade of comminution in the metaphyseal area. Jupiter et al. [9] reviewed 117 patients 2 years after surgery with a mean DASH of seven and a Gardland–Werley score of four together with the AO LCP study group. They saw a remaining intra-articular step-off of more than two mm in five out of 71 intra-articular fractures at the 2-year follow-up, but saw no changes in the radial length or radial angle of palmar tilt from the immediate postoperative X-ray to the final follow-up.

In our clinical follow-up, we could confirm the good to very good clinical results reported above: a loss of grip strength between the uninjured and injured side of <10 % might be the objective reason for the DASH of 5.6 place our results at the upper level of the results reported in the current literature. This is also true for weaker scores like the Gardland–Werley and Sarmiento scores only obtaining four different items including movement, pain, arthritis, and deformity. Radiologically, we had an immediate postoperative intra-articular step-off of less or more than 1 mm in 15 patients. We did not see any correlation with the clinical outcome of these patients. This was also true for arthritic changes observed 1 year after surgery. Furthermore, we saw secondary dislocation with a loss of radial length a mean of 2 mm, again without any effect on the clinical outcome. As suspected by others, the small amount of secondary dislocation might be due to the relative distal, almost subchondral screw position of the distal 2.2-mm fine-threaded miniscrews [23]. In reviewing our clinical results together with the radiological outcome, the reason for the minor influence of radiological deficiencies might be impossible to measure clinically, a fact that was also seen by others.

Despite the euphoria of the good and very good clinical results in addition to the lower complication rates of the volar plate system compared to an external fixation technique [24–26], surgeons must keep in mind that the use of this implant may cause serious complications: tendon irritation to rupture is a major problem with a rate of 0 to 38 % [8] reported in the current literature. Flexor tendon irritation to rupture is due to very distal plate positioning, whereas extensor problems are the consequence of a screw too long in length [8]. As we had a high incidence of very distal osteoporotic fractures and did not use other implants throughout the duration of this study, we found adequate soft tissue coverage of even the very low profile plate to be important. Although the pronator quadratus muscle might often be insufficient to cover the implant completely, we found it large enough in most cases to cover at least the ulnar parts of the plate to prevent damage to the most radial positioned flexor pollicis longus tendon. The orthopedic and trauma surgeon’s technique of intraoperative observation of the flexor pollicis longus tendon during thumb movement might be the reason for the relatively low incidence of one patient with flexor irritation, even in distal plate application.

Dorsally prominent screw tips have been identified as a cause of extensor tendon injuries: Beson et al. [27] reported screw penetration into the third dorsal compartment and fracture-related bony spurs or gapping at the fracture side as potential causes of the extensor pollicis longus tendon (EPL). We saw a total of four EPL problems: one patient had an initial traumatic rupture of the EPL tendon that was repaired during the first operation; two of our patients reported an insufficient thumb elevation in a period of 2–16 weeks after surgery. Here, prominent screw tips have been indentified as the reason for this problem. In a fourth patient with the same symptoms of thumb weakness, an early revision of the EPL tendon could exclude an alteration due a postoperative problem. Although the EPL tendon is especially known to be a typical problem, paying high operative attention may lead to fewer problems. In contrast to our high incidence of this complication, others do report less to zero tendon problems [4, 9]. A continuous intraoperative fluoroscopy with respect to the complicated dorsal shape of the distal radius, especially in the region of Lister’s tubercle, in addition to an aggressive treatment or revision in the situation of tendon irritation might prevent the major complication of a delayed or missed EPL rupture necessitating tendon transfer. Besides these highly relevant tendon problems, we saw one screw break leading to a secondary revision. Other common problems as described by others [4, 8, 10] such as CRPS, nerve irritation, carpal tunnel syndrome, and wound infections were not seen in our series.

Outcome scores, typically performed in follow-up studies of distal radius fractures, are highly prone to subjective influence [18], and objective radiological outcome measurements often do not correlate with the clinical outcome [28]. Today, grip strength measurements are often performed in order to objectively document functional outcomes [4, 8].

Due to its high incidence, operative treatment for distal radius fractures is performed in almost every trauma center. Good and very good results using palmar locking T-plates have been published by many, but mainly by large institutions [9, 11]. A smaller institution, with an incidence of <30 distal radial fractures necessitating operative treatment per year, obtained the good clinical, functional, and radiological results presented in this study. That fact that our results were obtained using a single implant for different fracture patterns supports the idea that a palmar locked angle implant can be used independently of the fracture type—for extra-articular as well as for comminuted intra-articular fractures. This may lead to a simplified treatment algorithm with a single implant for almost every distal radial fracture needing operative intervention. According to our results, it would generally not be necessary to refer displaced, distal radial fractures to a high-volume center.
